# High mobility group box 1 (HMGB1) mediates nicotine-induced podocyte injury

**DOI:** 10.3389/fphar.2024.1540639

**Published:** 2025-01-07

**Authors:** Sayantap Datta, Mohammad Atiqur Rahman, Saisudha Koka, Krishna M. Boini

**Affiliations:** ^1^ Department of Pharmacological and Pharmaceutical Sciences, College of Pharmacy, University of Houston, Houston, TX, United States; ^2^ Department of Pharmaceutical Sciences, Irma Lerma College of Pharmacy, Texas A&M University, Kingsville, TX, United States

**Keywords:** podocytes, HMGB1, nicotine, TLR4, smoking

## Abstract

**Introduction:**

Cigarette smoking is a well-established risk factor for renal dysfunction. Smoking associated with renal damage bears distinct physiological correlations in conditions such as diabetic nephropathy and obesity-induced glomerulopathy. However, the cellular and molecular basis of such an association remains poorly understood. High mobility group box 1(HMGB1) is a highly conserved non-histone chromatin associated protein that largely contributes to the pathogenesis of chronic inflammatory and autoimmune diseases such as sepsis, atherosclerosis, and chronic kidney diseases. Hence, the present study tested whether HMGB1 contributes to nicotine-induced podocyte injury.

**Methods and Results:**

Biochemical analysis showed that nicotine treatment significantly increased the HMGB1 expression and release compared to vehicle treated podocytes. However, prior treatment with glycyrrhizin (Gly), a HMGB1 binder, abolished the nicotine-induced HMGB1 expression and release in podocytes. Furthermore, immunofluorescent analysis showed that nicotine treatment significantly decreased the expression of podocyte functional proteins- podocin and nephrin as compared to control cells. However, prior treatment with Gly attenuated the nicotine‐induced nephrin and podocin reduction. In addition, nicotine treatment significantly increased desmin expression and cell permeability compared to vehicle treated podocytes. However, prior treatment with Gly attenuated the nicotine-induced desmin expression and cell permeability. Mechanistic elucidation revealed that nicotine treatment augmented the expression of toll like receptor 4 (TLR4) and pre-treatment with Gly abolished nicotine induced TLR4 upregulation. Pharmacological inhibition of TLR4 with Resatorvid, a TLR4 specific inhibitor, also attenuated nicotine induced podocyte damage.

**Conclusion:**

HMGB1 is one of the important mediators of nicotine‐induced podocyte injury through TLR4 activation.

## 1 Introduction

Cigarette smoking is a well-established cause for varied physiological dysfunctions viz cancer, atherosclerosis, thrombogenesis and vascular occlusion (Salonen and Salonen, 1993; United States. Dept. of Health and Human Services. et al., 1983; Passarelli et al., 2016; Jacobs et al., 2015; Lv et al., 2024; Datta et al., 2024a). In fact, cigarette smoking is responsible for close to 8 million mortalities globally every year- which also includes around 1.3 million non-smokers exposed to second-hand smoke (Organization, 2024). Cigarette smoking induces sympathetic stimulation, functional alterations in the endothelium, smooth muscle cell proliferation and dysfunction of vascular tone regulators- all of which play a significant role towards the onset and progression of arterial damage (Cryer et al., 1976; Ross, 1993). However, the exact mechanism involved remains largely unclear.

Studies over the years have suggested that cigarette smoking contributes to significant hemodynamic alterations and culminates to renal dysfunction (Franek et al., 1996; Mühlhauser, 1994). Meta-analysis patient-based studies and mechanistic investigations reveal that cigarette smoking is a significant risk factor that leads to both acute and chronic kidney injury onset and worsens conditions viz diabetic nephropathy, glomerulosclerosis, glomerulonephritis, and obesity associated glomerulopathy over the long term (Ito et al., 2020; Ataka et al., 2023; Wang et al., 2021; McDermott et al., 2020; Pesce et al., 2021; Liao et al., 2019; Jaimes et al., 2021; Gündoğdu and Anaforoğlu, 2022). Cigarette smoke is an aerosol comprising of both vapour and particulate phase materials (Smith and Fischer, 2001; Thielen et al., 2008; Borgerding and Klus, 2005; Osborne et al., 1956). Vapor phase constituents chiefly include carbon monoxide, acetaldehyde, formaldehyde, and nitrogen oxides (Moldoveanu and Charles, 2007; Pang and Lewis, 2011). Nicotine, biologically one of the most stable and active components of the particulate phase, is central to most of the pathophysiological dysfunctions associated with cigarette smoking (Benowitz and Burbank, 2016; West, 2017; Schweitzer et al., 2015). Active or passive forms of nicotine exposure enhances renal oxidative stress through mitochondrial reactive oxygen species (ROS) upregulation, transcriptional activation of the pro-apoptotic and pro-oxidant p66shc in renal proximal tubule cells, and NLRP3 inflammasome activation (Arany et al., 2016; Wu et al., 2018; Singh et al., 2019a; Wu et al., 2020; Datta et al., 2024b). Oxidative stress drives inflammatory cascades and renal fibrosis and culminates to chronic kidney injury and end-stage renal diseases (ESRD) (Arany et al., 2016; Wu et al., 2018; Singh et al., 2019a; Ramalingam et al., 2019; Akkoyun and Karadeniz, 2016; Harwani et al., 2016; Zheng et al., 2020; Mishra et al., 2015; Jha et al., 2018; Mayyas and Alzoubi, 2019). Although nicotine has been identified as a major risk factor for the onset and progression of CKI, clear mechanistic understanding of nicotine induced kidney damage remains largely unclear.

High Mobility Group Box 1 (HMGB1) is a highly conserved non-histone chromatin-associated protein widely recognized for its regulatory impact on vital cellular processes like autophagy, apoptosis, and cell survival (Datta et al., 2024a; Narumi et al., 2015; Tang et al., 2010a; Singh et al., 2019b). HMGB1 exhibits dual functionality based upon its localization- as a non-histone protein in the nucleus and as a prototypic damage associated molecular pattern (DAMP) molecule upon extracellular release (Gazzar et al., 2009; Yu and Spring, 1977; Zhang et al., 2019; Andersson et al., 2018). Upon extracellular release, HMGB1 orchestrates inflammatory cascades, immunological responses and drives conditions viz cancer, neurodegeneration, and cardiovascular complications (Tang et al., 2010b; Festoff et al., 2016; Huang et al., 2016). Pathophysiological insights reveal that HMGB1 also plays a significant role in the onset and progression of kidney damage (Good et al., 2015; Ito et al., 2007). Extracellular HMGB1 drives cellular damage and inflammatory cascades in renal ischemic reperfusion injury (IRI) and plays a pivotal role in acute kidney injury (AKI) onset via Tumor Necrosis Factor (TNF)-α/HMGB1 inflammatory signalling (Wu et al., 2010; Wang et al., 2020). Hence, in the current study we tested whether HMGB1 mediates nicotine-induced podocyte injury.

## 2 Materials and methods

### 2.1 Cell culture

A conditionally immortalized murine podocyte cell line (Division of Nephrology, Department of Medicine, Mount Sinai School of Medicine, NY, United States) was cultured undifferentiated with 10 U/mL recombinant mouse interferon-γ at 33°C on collagen I-coated flasks in RPMI-1640 media (Thermo Fisher Scientific, United States) containing 10% foetal bovine serum (R&D Systems, United States), 100 U/mL penicillin and 100 mg/mL streptomycin (Life Technologies Corporation, NY, United States). The podocytes were allowed to differentiate at 37°C for 10–14 days without interferon–γ. These differentiated podocytes were subsequently utilized for the experiments (Koka et al., 2019). Podocytes were pretreated with Glycyrrhizin (Gly, 120 μM) (Tokyo Chemical Industry Co. Ltd., Tokyo, Japan; dissolved in water for 30 min) (Mollica et al., 2007; Palumbo et al., 2004) or Resatorvid (TAK-242; 100 nM, Med Chem Express; United States; dissolved in dimethyl sulfoxide for 15 min) (Kashani et al., 2020; Kashani et al., 2019; Matsunaga et al., 2011) prior to nicotine treatment for overnight (8 μM; dissolved in water) (Singh et al., 2019a).

### 2.2 Immunofluorescence staining

The podocytes were grown on eight-well chamber slides (Thermo Fisher Scientific, United States) and treated as experimentally designed. The cells were then fixed with 4% paraformaldehyde for 15 min. Cells were then washed in phosphate-buffer saline (PBS) followed by blocking with 1% bovine serum albumin (BSA) for 1 h at room temperature. This was followed by primary antibody incubation at 4°C overnight against podocin (1:200, Sigma-Aldrich, United States; catalogue number P0372), desmin (1:200, Abcam, Cambridge, CA, United States; catalogue number ab15200), HMGB1 (1:200, Abcam, Cambridge, CA, United States; catalogue number ab18256) and nephrin (1:200; Santa Cruz Biotechnology, Inc., United States; catalogue number sc-377246). Subsequently, the slides were incubated at room temperature with Alexa Fluor 555-labeled secondary antibody (1:500, Invitrogen; catalogue numbers A32732 and A-31570) for 1 h. The slides were washed with PBS and mounted with DAPI containing mounting medium (Vector Laboratories, Inc., United States). The slides were sequentially scanned and imaged using confocal microscopy (Leica SP8 STED Confocal Microscope). ImageJ software was used for the quantification of mean fluorescence intensity of the images developed and statistical analysis was done using GraphPad Prism 9.2.0.

### 2.3 Extracellular HMGB1 quantification

The cultured murine podocytes were treated with nicotine with and without Gly (120 µM) overnight. The supernatants were collected and the concentration of the released extracellular HMGB1 was measured using commercial enzyme-linked immunosorbent assay (ELISA) kit (MyBioSource, San Diego, CA, United States) as per the manufacturer’s instructions.

### 2.4 Cell permeability assay

The monolayer permeability of podocytes in culture was measured according to previously optimized method (Boini et al., 2018; Boini et al., 2010). In summary, the podocytes were seeded in the upper chambers of 0.4 μm polycarbonate trans-well filters of a 24-well filtration microplate (Corning Inc., United States). After optimum confluence, the culture medium was replaced with fresh serum free RPMI 1640 media in presence of nicotine (8 μM) with or without Gly (30 min pretreatment) or Resatorvid (15 min pretreatment) and incubated overnight. Subsequently, the serum free media was discarded, fresh phenol red-free RPMI-1640 with 70 kDa fluorescein isothiocyanate (FITC)-dextran (2.5 μM) was added in the upper chambers and incubated for 3 h. Then, the filtration microplate was removed and the medium in the lower compartment was collected. The fluorescence intensity was measured in a spectrofluorometer (BioTek Instruments, Inc., Winooski, VT, United States) at 494 nm excitation and 521 nm emission wavelengths. The relative permeable fluorescence intensity was used as a measure of cell permeability.

### 2.5 Western blot

After being treated as experimentally designed, the cultured podocytes were washed with ice-cold PBS twice followed by homogenization in cell lysis buffer (BioVision, United States). After homogenization, they were centrifuged at 1,500 × *g* for 15 min at 4°C. The supernatants were collected and stored at −80°C until use. Cell homogenates were denatured with reducing Laemmli sodium dodecyl sulphate (SDS)-sample buffer and boiled for 5 min at 95°C. Homogenates were run on SDS-PAGE gel, transferred into a polyvinylidene difluoride (PVDF) membrane (Thermo Fisher Scientific, United States), and blocked with 5% BSA. The membranes were probed with primary antibodies for podocin (1:1000, Sigma-Aldrich, United States; catalogue number P0372), TLR4 (1:1000, Santa Cruz Biotechnology, Inc., United States; catalogue number sc-293072), RAGE (1:1000; Sigma-Aldrich, United States; catalogue number R5278), TLR2 (1:1000; Santa Cruz Biotechnology, Inc., United States; catalogue number sc-21759),and β-actin (1:1000; Santa Cruz Biotechnology, Inc., United States; catalogue number sc-47778) overnight at 4°C. The membranes were subsequently washed with 1X tris-buffered saline (TBS) and 0.5% tween, incubated with secondary antibody (catalogue numbers sc-2357 and 1706516) for 1 h, and then conjugated to horseradish peroxidase (HRP)-labelled immunoglobulin G. The bands on the membrane were enhanced by chemiluminescence. The membranes were scanned using Licor chemiluminescence system.

### 2.6 Statistical analysis

Quantification data for all the experiments were analysed using GraphPad Prism 9.2.0. Data was plotted as arithmetic mean ± standard error of mean (SEM); *n* represents the number of independent experiments. All data were tested for significance using Student’s unpaired t-test or one way ANOVA followed by a *post hoc* test. Results with p < 0.05 were considered statistically significant.

## 3 Results

### 3.1 Nicotine upregulates expression and extracellular release of HMGB1 in podocytes

**FIGURE 1:**
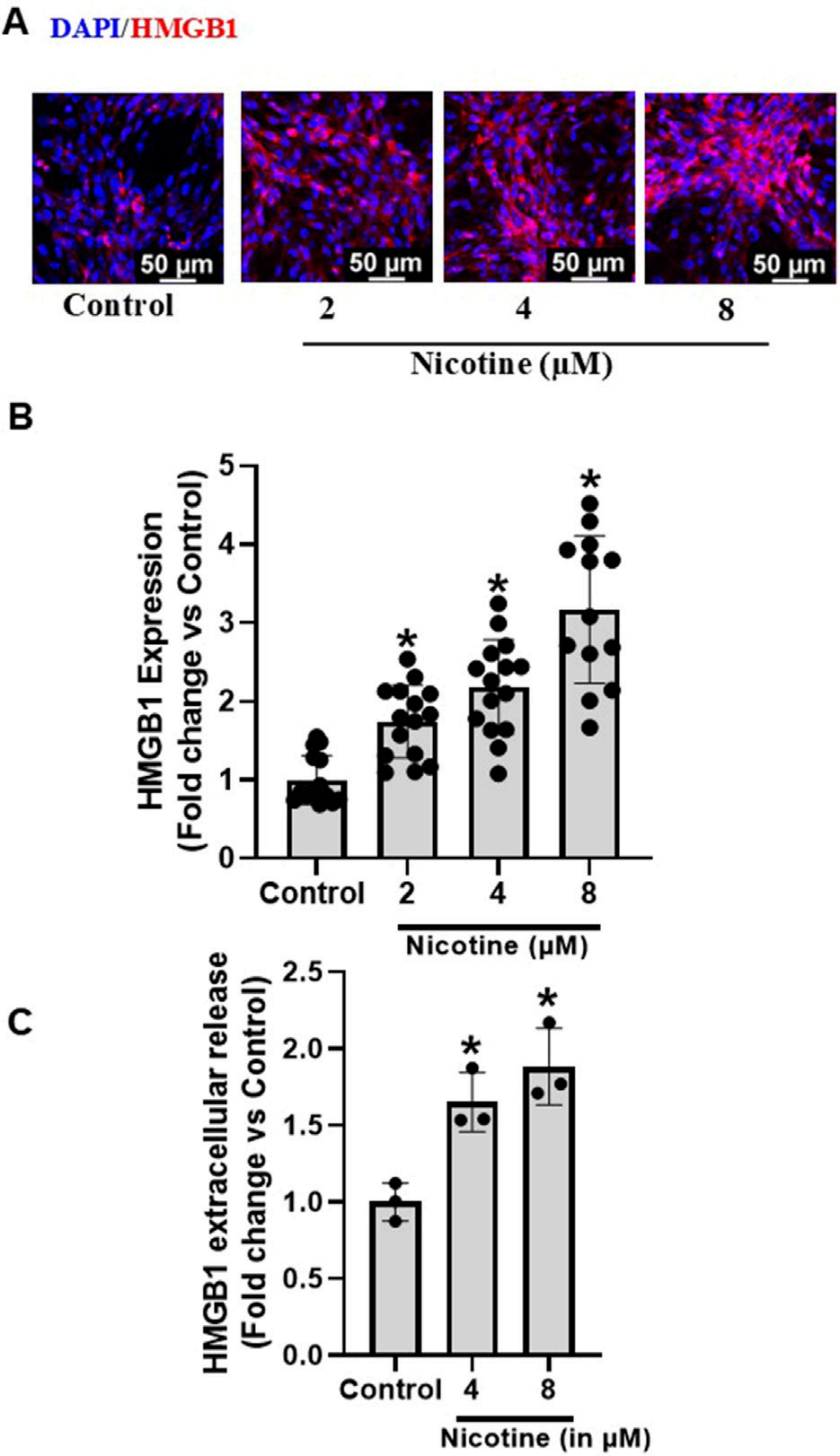
Effect of Nicotine on expression and extracellular release of HMGB1 in murine podocytes. Representative immunofluorescence images (Scale- 50 µm) **(A)** and summarized quantification data **(B)** depict HMGB1 expression in podocytes subjected to increasing concentrations of nicotine. ELISA quantification data **(C)** shows extracellular release pattern of HMGB1 as influenced by increasing nicotine concentrations. The immunofluorescence images were quantified using ImageJ software and statistical analysis of the quantified data was accomplished using GraphPad Prism 9.2.0. *p < 0.05 vs. control group.

To investigate the influence of nicotine on HMGB1 expression and extracellular release in podocytes, the cultured murine podocytes were treated with increasing concentrations of nicotine for 16 h. Our results show that nicotine dose dependently increases the HMGB1 expression (Figures 1A, B) and HMG1 extracellular release (Figure 1C) in podocytes.

### 3.2 HMGB1 targeted inhibition attenuates nicotine-induced HMGB1 expression and extracellular localization

**FIGURE 2:**
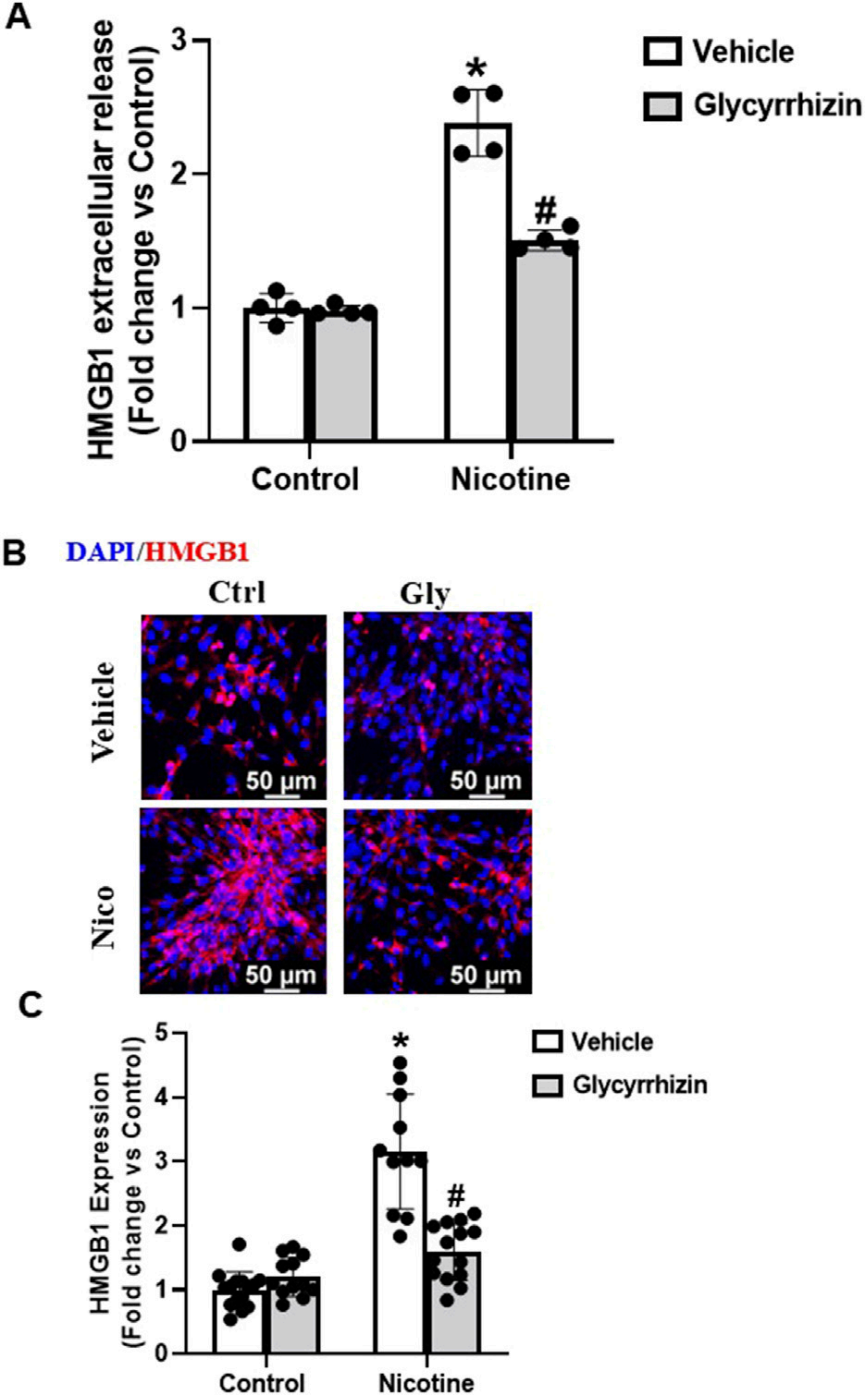
HMGB1 specific inhibition attenuates nicotine induced HMGB1 expression and extracellular release. ELISA quantification data **(A)**, representative immunofluorescence images (Scale- 50 µm) **(B)** and summarized quantification **(C)** outlines the influence of Gly (HMGB1 specific binder) on nicotine induced extracellular release and expression of HMGB1. The immunofluorescence images were quantified using ImageJ software and statistical analysis of the quantified data was accomplished using GraphPad Prism 9.2.0. *p < 0.05 vs. control group, #p < 0.05 vs. nicotine treated group; Ctrl- Control, Gly- Glycyrrhizin, Nico- Nicotine.

Studies over the years have identified Gly, obtained from liquorice (*Glycyrrhiza glabra*) plant, as a potent inhibitor of HMGB1 and associated pro-inflammatory cascades (Mollica et al., 2007; Palumbo et al., 2004). Herein, we tested the influence of Gly on nicotine-induced HMGB1 expression and extracellular release. Our investigations reveal that Gly attenuates nicotine induced increase in extracellular release of HMGB1 (Figure 2A). Additionally, our immunofluorescence results show that Gly attenuates nicotine induced HMGB1 expression upregulation (Figures 2B, C). Together, these results confirm that HMGB1 binder Gly interferes with and attenuates nicotine induced increase in HMGB1 expression and extracellular release.

### 3.3 Nicotine mediates podocyte damage in a HMGB1-dependent manner

**FIGURE 3:**
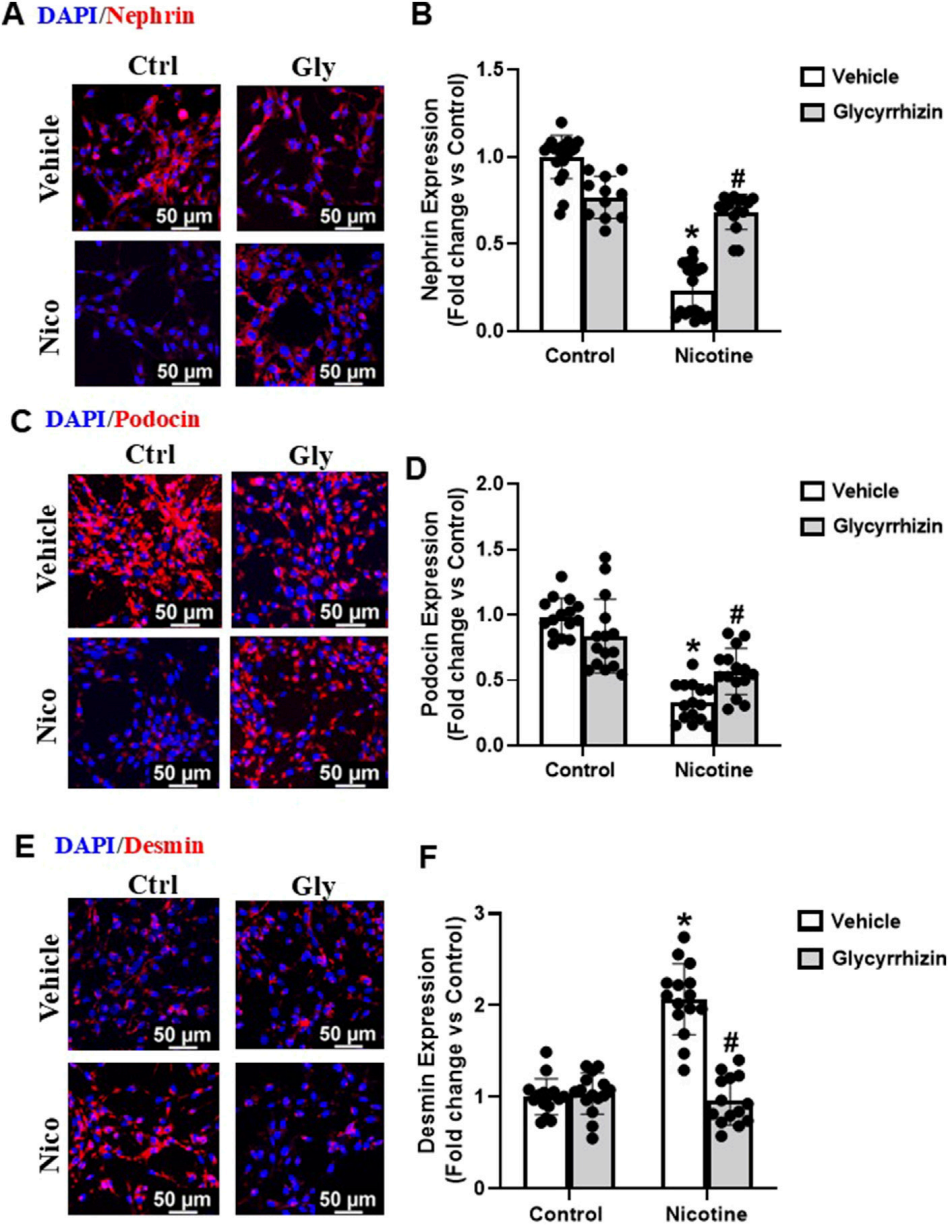
HMGB1 specific inhibition attenuates nicotine induced functional and structural damage in podocytes. Representative immunofluorescence images (Scale- 50 µm) and summarized quantification outlines the influence of Gly on nephrin **(A, B)**, podocin **(C, D)** and cytoskeletal desmin **(E, F)** expression in podocytes treated with nicotine. ImageJ software was used for quantification of the immunofluorescence images and statistical analysis of the quantified data was accomplished using GraphPad Prism 9.2.0. *p < 0.05 vs. control group, #p < 0.05 vs. nicotine treated group; Ctrl- Control, Gly- Glycyrrhizin, Nico- Nicotine.

Podocyte specific proteins podocin and nephrin are central to podocyte function and are downregulated upon podocyte damage (Saleem et al., 2002; Perico et al., 2016). In coherence with our former investigations (Singh et al., 2019a), our immunofluorescence analysis showed that nicotine-induced decrease in nephrin and podocin expression (Figures 3A–D). However, pre-treatment with Gly attenuated nicotine induced nephrin and podocin downregulation (Figures 3A–D). In addition, our immunofluorescence analysis studies also reveal that nicotine upregulates cytoskeletal desmin levels and mediates podocyte damage (Figures 3E, F). However, prior treatment with Gly prevents nicotine induced desmin upregulation (Figures 3E, F). Our results confirm that nicotine drives podocyte damage through HMGB1 activation and prior attenuation of HMGB1 extracellular release protects against nicotine induced podocyte injury.

### 3.4 HMGB1 targeted inhibition attenuates nicotine induced podocyte permeability

**FIGURE 4:**
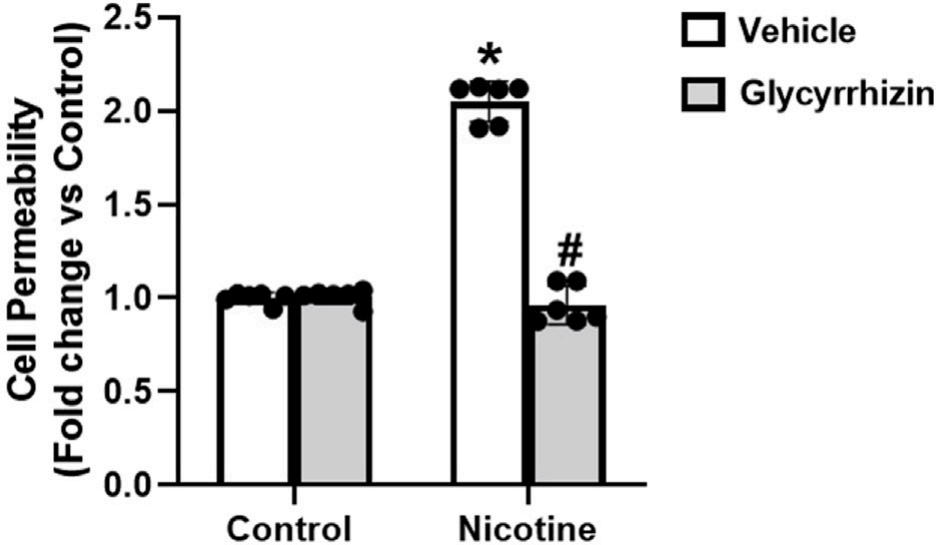
HMGB1 inhibition attenuates nicotine induced podocyte permeability. The influence of Gly on nicotine induced podocyte permeability was assessed using cell permeability assay. Statistical analysis of the quantified data was accomplished using GraphPad Prism 9.2.0. *p < 0.05 vs. control group, #p < 0.05 vs. nicotine treated group.

Next, in functional studies we tested how monolayer permeability of podocytes was affected by nicotine in the presence and absence of Gly. Nicotine augments podocyte permeability relative to their control cells (Figure 4). However, prior treatment with Gly attenuates nicotine induced podocyte permeability upsurge (Figure 4). Our results confirm that inhibition of HMGB1 nucleus/cytoplasm translocation prevents nicotine associated upsurge in podocyte permeability.

### 3.5 HMGB1 inhibition prevents nicotine induced toll-like receptor (TLR)4 upregulation in podocytes

**FIGURE 5:**
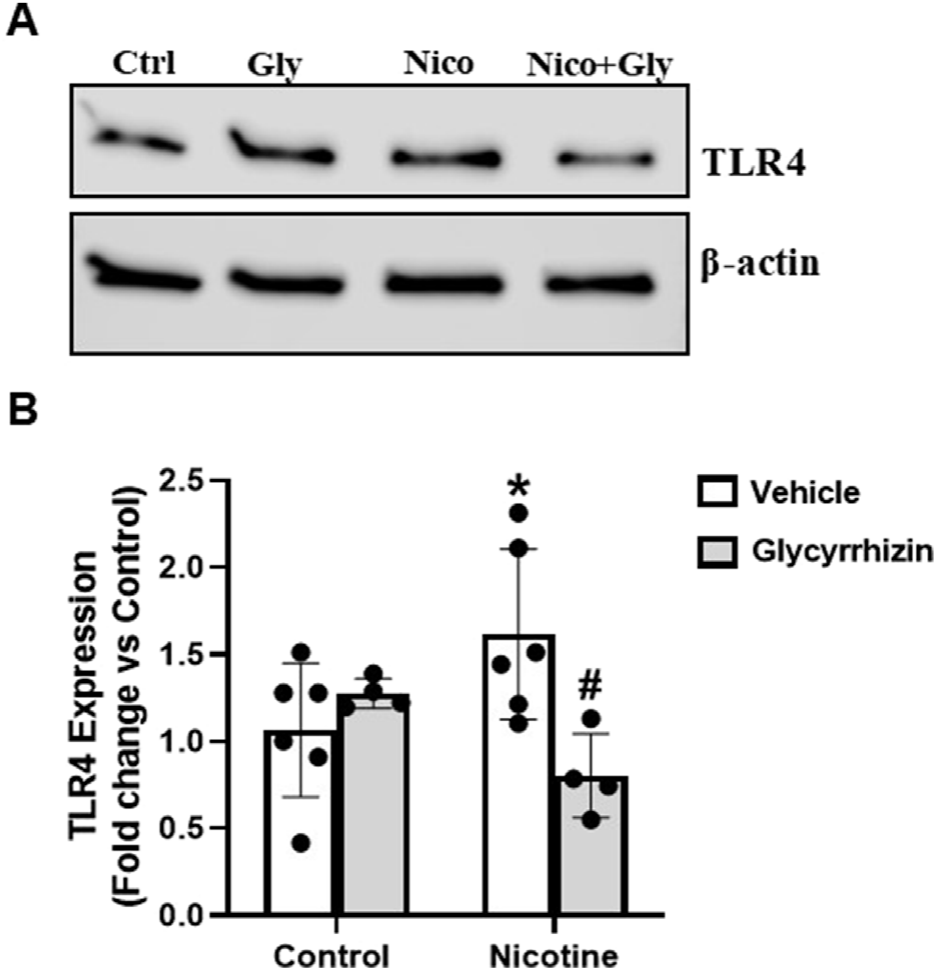
HMGB1 inhibition attenuates nicotine induced Toll-like receptor (TLR)-4 upregulation in podocytes. Western blot images **(A)** and associated quantification data **(B)** summarize the influence of Gly on nicotine induced TLR4 upregulation in podocytes. Western blot band intensity values were obtained using Image Studio Lite 5.2 and statistical analysis of the quantified data was done using GraphPad Prism 9.2.0. *p < 0.05 vs. control group, #p < 0.05 vs. nicotine treated group; Ctrl- Control, Gly- Glycyrrhizin, Nico- Nicotine, Nico + Gly- Nicotine + Glycyrrhizin.

Further we tested the influence of nicotine on cultured podocytes with and without HMGB1 inhibition. Our results reveal that nicotine increases TLR4 expression in podocytes as compared to the control counterparts (Figures 5A, B). However, prior treatment with Gly attenuated nicotine induced TLR4 upsurge (Figures 5A, B). We also tested for the influence of nicotine on TLR2 and receptor for advanced glycation end products (RAGE) in the presence and absence of Gly. TLR2 and RAGE levels exhibited no significant difference for nicotine with or without Gly relative to control podocytes (data not shown here). These results outline that HMGB1 mediates nicotine induced podocyte damage potentially via pro-inflammatory TLR4 upregulation and HMGB1 inhibition attenuates nicotine- induced TLR4 upregulation.

### 3.6 TLR4 pharmacological inhibition lessens nicotine induced podocyte damage

**FIGURE 6:**
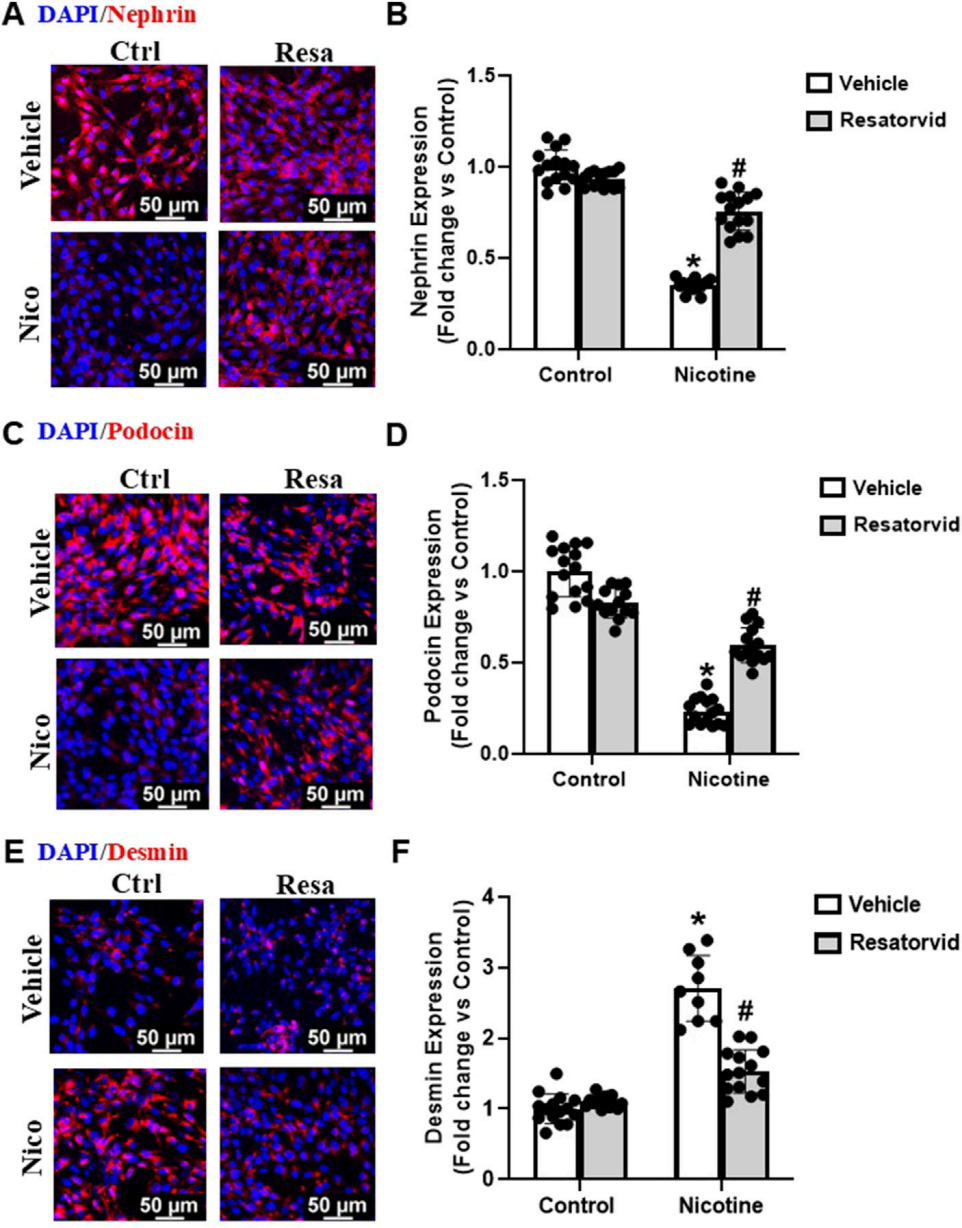
Pharmacological TLR4 inhibition lessens nicotine associated functional and structural injury in podocytes. Representative immunofluorescence images (Scale- 50 µm) and summarized quantification outlines the influence of Resatorvid (TLR4 specific inhibitor) on nephrin **(A, B)**, podocin **(C, D)** and desmin **(E, F)** expression in podocytes treated with nicotine. ImageJ software was used for quantification of the immunofluorescence images and statistical analysis of the quantified data was accomplished using GraphPad Prism 9.2.0. *p < 0.05 vs. control group, #p < 0.05 vs. nicotine treated group; Ctrl- Control, Resa- Resatorvid (100 nM), Nico- Nicotine (8 µM).

In this study, we utilized Resatorvid (TAK-242), a small molecule inhibitor of TLR4 (Kashani et al., 2020; Kashani et al., 2019; Matsunaga et al., 2011), to investigate its influence on nicotine-induced podocyte injury. Dose-dependent studies were carried out to determine the optimum concentration of Resatorvid for our experiments (data not shown). 100 nM was found to be the optimum concentration for Resatorvid and utilized for further experiments in this study. Our immunofluorescence studies showed that Resatorvid protects against nicotine associated decrease in nephrin and podocin levels (Figures 6A–D). Additionally, Resatorvid attenuates nicotine induced desmin upregulation (Figures 6E, F). Together, these results confirm that nicotine-induced podocyte damage is mediated via TLR4 activation and TLR4 specific inhibition protects against nicotine associated podocyte damage.

### 3.7 TLR4 specific inhibition attenuates nicotine induced podocyte permeability

**FIGURE 7:**
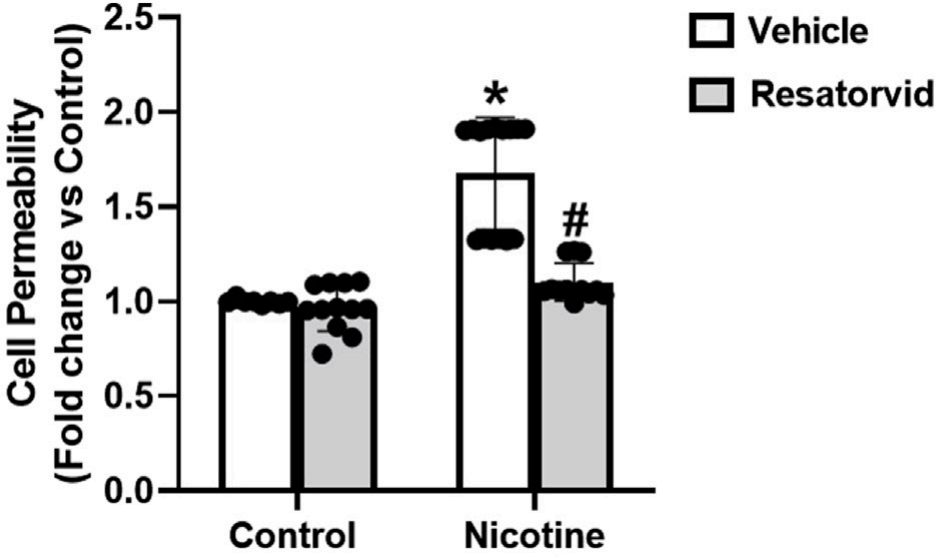
Pharmacological TLR4 inhibition attenuates nicotine induced podocyte permeability. The impact of Resatorvid on nicotine induced podocyte permeability was assessed using cell permeability assay. Statistical analysis of the quantified data was accomplished using GraphPad Prism 9.2.0. *p < 0.05 vs. control group, #p < 0.05 vs. nicotine treated group.

Furthermore, we tested the influence of Resatorvid mediated TLR4 inhibition on nicotine induced upsurge in podocyte permeability. Our results show that TLR4 inhibition protects against nicotine associated rise in podocyte monolayer permeability (Figure 7). These results further consolidate our understanding that TLR4 inhibition protects against nicotine-induced podocyte damage.

## 4 Discussion

The goal of the present study is to determine whether HMGB1 is implicated in the development of nicotine-induced podocyte injury. Our findings demonstrate that nicotine upregulates TLR4 expression and HMGB1 expression and extracellular release in mediating podocyte injury. However, pre-treatment with HMGB1 inhibiting Gly attenuated nicotine induced HMGB1 and TLR4 upregulation and associated podocyte damage. To our understanding, this is the first study to establish the pathophysiological role of HMGB1 in nicotine induced podocyte injury, primarily via TLR4 upregulation.

Nicotine, one of the most stable and active components of cigarette smoke, plays a pivotal role towards the onset and progression of proteinuria, diabetic nephropathy and subsequently, CKD (Briganti et al., 2002; Rossing et al., 2002; Hallan and Orth, 2011). Chronic exposure to nicotine augments mitochondrial ROS levels and exacerbates mitochondrial depolarization and renal cell apoptosis and/or necrosis- chiefly through the inhibition of epidermal growth factor receptor/Ras/Mitogen activated protein kinase (MAPK)/Extracellular signal-regulated kinase (ERK) cascade (Arany et al., 2010; Arany et al., 2008). Nicotine augments cyclooxygenase (COX)-2 expression and ERK1/2 phosphorylation and mediates proliferation and fibronectin generation in kidney-derived mesangial cells (Jaimes et al., 2009; Hua et al., 2010). Nicotine induced oxidative stress upregulates c-Jun N-terminal kinase (JNK) driven activator protein (AP)-1 activation and attributes to the tubular effects of nicotine (Arany et al., 2011). Renal function evaluation studies reveal that nicotine significantly reduces renal plasma flow rate and augments microalbuminuria risk (Gambaro et al., 1998; Gerstein et al., 2000). Smoking associated nicotine exposure downregulates estimated glomerular filtration rate (eGFR) and augments progression of proteinuria and autosomal polycystic kidney disease (Ozkok et al., 2013; Chase et al., 1991). However, exact mechanistic understanding of nicotine induced renal damage remains poorly understood and largely limits draggability in this regard.

High Mobility Group Box 1 (HMGB1) is a highly conserved non-histone chromatin-associated protein across species (Gazzar et al., 2009; Štros, 2010). It functions as a non-histone protein in the nucleus and as an inducer of inflammatory cytokines upon extracellular release (Gazzar et al., 2009; Andersson et al., 2018; Genschel and Modrich, 2009). Existing paradigm of studies show that HMGB1 is central to the onset and progression of renal dysfunctions (Poston and Koyner, 2019; Wang et al., 2004; Zhao et al., 2020). Studies outline that HMGB1 exhibits a regulatory role in driving onset and progression of secondary renal damage like glomerulonephritis, diabetic nephropathy and lupus nephritis (Tachibana et al., 2019; Andersen et al., 2014). Extracellularly released HMGB1 activates NF-ƘB signalling and mediates release of pro-inflammatory cytokines like TNF-α, Interleukin (IL)-6 and IL-1β in serum (Anders et al., 2018; Chen et al., 2018; Shen et al., 2024). However, the pathophysiological role of HMGB1 in smoking associated renal damage remains poorly understood. In this regard, our present study confirms that nicotine augments intracellular expression and extracellular release of HMGB1 in murine podocytes in a dose-dependent manner. Prior treatment with Glycyrrhizin (Gly), a HMGB1 binder (Mollica et al., 2007; Palumbo et al., 2004), attenuates nicotine associated HMGB1 upregulation in podocytes.

Podocin and Nephrin are podocyte specific proteins that are central to structural and functional integrity maintenance in podocytes (Saleem et al., 2002; Perico et al., 2016). Podocin governs structural organization of the slit diaphragm via interaction with podocyte specific nephrin and CD2 associated protein (CD2AP) (Saleem et al., 2002; Huber et al., 2003; Schwarz et al., 2001). On the other hand, nephrin is central to podocyte maturation during glomerular development and development of the slit diaphragm junctional complex (Done et al., 2008; Li et al., 2015). Our results confirm that nicotine downregulates podocin and nephrin expression and mediates podocyte damage. However, pre-treatment with HMGB1 binder Gly prevents nicotine induced podocin and nephrin downregulation. Cytoskeletal protein desmin is central to intermediate filament formation and maintenance of structural and mechanical integrity of podocytes (Schell and Huber, 2017a; Nagata, 2016). Dysregulation of the podocyte cytoskeletal framework attributes to anomalies chiefly foot process retraction and proteinuria (Fuchshofer et al., 2011; Schell and Huber, 2017b). In fact, upregulation of desmin constitutes one of the key features of podocyte injury associated glomerular diseases (Zou et al., 2006). Our investigations show that nicotine upregulates desmin expression in mediating podocyte damage. However, prior Gly treatment attenuates nicotine induced desmin upsurge. To further assess the functional significance of HMGB1 in nicotine induced podocyte injury, we examined the impact of nicotine on podocyte permeability with and without Gly treatment. Our results showed that nicotine augments podocyte permeability to FITC-dextran through HMGB1 activation and this was prevented via prior treatment with HMGB1 inhibitor Gly. These findings postulate that nicotine induced structural and functional decadence in podocytes potentially occurs via HMGB1 activation.

TLR4, a member of pattern recognition receptor (PRR) family, is central to intrarenal inflammatory response initiation- chiefly characterized by increase in proinflammatory cytokine and chemokine expression, neutrophil and monocyte influx and urinary elimination of cytokines and chemokines (Ramesh and Reeves, 2002; Ramesh et al., 2007; Ramesh and Reeves, 2003; Majumder et al., 2024). The cytokine-like proinflammatory properties of HMGB1 have been established to be primarily TLR4 dependent (Kim et al., 2013). HMGB1, upon activation, interacts with TLR4/myeloid differentiation protein 2 (MD2) which engages coreceptor CD14 (Yang et al., 2015; He et al., 2018). This, in turn, promotes the release of monocyte chemoattractant protein (MCP)-1, IF-induced protein 10 (IP-10) and macrophage inflammatory protein 1α (MIP-1α) (Nano et al., 2013; Rabadi et al., 2012). Pharmacological inhibition of TLR4 and/or associated pro-inflammatory signalling has been vastly undertaken to better understand the signalling mechanisms driving kidney damage (Niu et al., 2019; González-Guerrero et al., 2017; Shi et al., 2017). Existing paradigm of studies establish a strong correlation between HMGB1 activation and TLR4 signalling. However, it remains unknown whether TLR4 signalling is involved in nicotine induced renal damage. Our study confirms that nicotine upregulates TLR4 levels and drives podocyte damage. However, prior treatment with HMGB1 inhibiting Gly attenuates nicotine induced TLR4 upregulation. These findings postulate that nicotine upregulates TLR4 levels in podocytes potentially via HMGB1 activation. To further validate our hypothesis, we advocated pharmacological inhibition of TLR4 using Resatorvid to investigate nicotine-induced podocyte damage from functional and structural perspectives. Resatorvid mediated TLR4 inhibition recovers podocin and nephrin downregulation induced by nicotine associated podocyte damage. TLR4 inhibition also attenuates nicotine associated upregulation of desmin expression and podocyte permeability. Together, these findings confirm that HMGB1 activation and extracellular release drives TLR4 signalling and mediates nicotine induced podocyte injury.

In conclusion, our results show that HMGB1 is an important mediator of nicotine induced podocyte damage potentially via TLR4 activation. The amelioration of podocyte injury by inhibition of HMGB1 during nicotine stimulation implicates the pivotal role of HMGB1 in smoking-induced podocyte injury.

## Data Availability

The raw data supporting the conclusions of this article will be made available by the authors, without undue reservation.
